# Heterogeneity of demoralization syndrome in Chinese cancer patients: an optimal cut-off threshold for the demoralization scale

**DOI:** 10.3389/fpsyt.2025.1560247

**Published:** 2025-12-17

**Authors:** Yanhua Li, Xiaoxin Liu, Qun Yang

**Affiliations:** 1 Department of Psychiatry, The Second Xiangya Hospital, Central South University, Changsha, China; 2 Clinical Nursing Teaching and Research Section, Second Xiangya Hospital, Central South University, Changsha, China; 3 Department of Oncology, Second Xiangya Hospital, Central South University, Changsha, China; 4 Intravenous Therapy Center of Hunan Province, Changsha, China

**Keywords:** cancer, cut-off threshold, demoralization, demoralization scale, heterogeneity, profile

## Abstract

**Background:**

The development of numerous demoralization assessment tools has significantly contributed to the field, with the Demoralization Scale being the most frequently utilized. Nevertheless, the inconsistency in cutoff values for these tools hinders accurate estimation of demoralization, underscoring the need for research to determine an optimal threshold. This study examined the heterogeneity of demoralization syndrome among cancer patients in China, aiming to establish an optimal cut-off value for the mandarin version demoralization scale.

**Method:**

A cross-sectional study recruited 971 cancer patients from Hunan Province between June 14, 2022 and June 13, 2023. Latent profile analysis was used to identify distinct profiles, and receiver operating characteristic curve analysis determined the optimal cut-off point.

**Result:**

Three distinct profiles were identified: “minimal demoralization - meaningful” (22.3%), “moderate demoralization” (59.9%), and “severe demoralization - hopelessness” (17.9%). The mandarin version demoralization scale showed high accuracy (AUC = 0.995) with an optimal cut-off point of 23.5 (sensitivity: 98.9%, specificity: 94.8%, and accuracy: 93.7%).

**Conclusion:**

The distinct profiles highlight varying demoralization syndrome in cancer patients. Notably, the Mandarin Version Demoralization Scale exhibits excellent properties, with a cutoff of 23.50 for Chinese cancer patients. Our study deepens understanding, offering insights for standardized clinical classifications, enabling cross-setting comparisons.

## Introduction

To date, cancer mortality has declined by 29% (2.9 million fewer deaths), according to the WHO, but cancer still poses a significant threat to human lives. In 2020, nearly 19.3 million new cases and 10 million deaths were reported globally (1). The prevalence of cancer and the growing number of cancer survivors have led to increased attention to patients’ psychological issues (2).

Demoralization syndrome, as a unique existence which is different from depression and anxiety (3), resulting from an individual’s inability to adapt to long-term stress, is characterized by persistent pain, helplessness, hopelessness, a sense of meaninglessness, feelings of incompetence, and decreased self-esteem (4). It is highly prevalent in cancer patients, with an incidence rate ranging from 23.7% to 88.8% (3–7). However, it is often overlooked in clinical practice and misunderstood as depression, delaying proper support leading and leading to significant psychological distress for patients. This, in turn, affects the treatment and prognosis of this psychological condition to varying degrees, and may even trigger suicidal ideation or behaviors (8, 9). After reflecting on the past half century of progress, the Demoralization Syndrome has transformed from a solitary psychosomatic research variable into a more comprehensive diagnostic framework. This syndrome has been assigned a code in the latest International Classification of Diseases (ICD-11) as a distinct psychological condition, though it is not currently listed as a standalone diagnosis in the Diagnostic and Statistical Manual of Mental Disorders (DSM-5). Consequently, it has garnered increasing attention from scholars both domestically and internationally. The clinical diagnosis of demoralization requires the Structured Interview for the Diagnostic Criteria for Psychosomatic Research (DCPR). However, due to the need for specialized personnel, cumbersome procedures, time-consuming nature, and high costs, it is not feasible to apply this method to screening a large population of cancer patients in clinical settings.

Over the past few decades, various self-report forms of demoralization assessment tools have been developed, such as the Psychiatric Epidemiology Research Interview-Demoralization (PERI-D), the Subjective Incompetence Scale (SIS), the Demoralization Scale (DS) and its simplified version (Demoralization Scale II, DS-II), and the Short Demoralization Scale (SDS). Among them, the DS was developed by Kissane et al. (10) and is currently the most commonly used tool for assessing demoralization. It has been widely applied in multiple countries and regions, including China (using the 24-Item Mandarin Version of Demoralization Scale) (3, 11), and in various cancer patient populations, with satisfactory reliability and validity, and can effectively distinguish demoralization from depression (2, 11). Previous studies have often relied on the original study (10), using a total score of >30 or ≥30 points as the cutoff value for DS (3, 8, 10). Some scholars have also adopted a cutoff value of ≥36 points (1.5 times the total score when each item is scored 1 point) as the criterion for clinical high-level demoralization (12). Alternatively, mean ± standard deviation (SD) has been also used to classify clinical high, medium, and low levels of demoralization (i.e., low: Score<mean-SD, medium: mean-SD≤ Score ≤mean+ SD, high: Score >mean+ SD, with the high-level demoralization group considered to represent clinical demoralization) (9). This existing deviation may lead to an inaccurate estimation of demoralization, impacting the identification of key risk factors and resulting in inaccurate or even incorrect outcomes. This methodological variation may introduce measurement error, affecting the accuracy of demoralization symptom estimates and, in turn, the identification of associated risk factors. Therefore, it is essential to conduct research to determine the optimal cut-off threshold for Demoralization Scale.

Latent profile analysis (LPA) detects unobserved heterogeneity in individuals’ responses to continuously observed variables, clustering them into homogeneous subgroups. Despite its semi-subjective nature, LPA has a low misclassification rate (13) and enables classifying disease states without a clinical gold standard. This approach aids in identifying case groups, determining optimal evaluation thresholds, and estimating sensitivity/specificity (14).

To address the identified research gaps, this study seeks to explore the heterogeneity and potential subgroups of demoralization syndrome among Chinese cancer patients via LPA, and establish an optimal screening threshold using the 24-item Mandarin Version Demoralization Scale. Aimed at addressing the unmet need for standardized clinical screening, this research further intends to validate this threshold for patients with Stage II-IV cancer, thereby providing a targeted tool for early identification of demoralization and enriching understanding of its clinical presentation. To this end, this study addresses two core research questions: (1) What latent subgroups of demoralization can be identified using LPA among Chinese cancer patients, and how can these subgroups be characterized? (2) To what extent can the identified subgroup structure support the derivation of an empirically grounded, clinically meaningful cutoff for demoralization screening?

## Materials and methods

### Design and sampling

This research followed an explorative cross-sectional study design. The STROBE guideline was utilized to report data (15). A convenience sample method was carried out for the recruitment of cancer patients from the oncology wards of two comprehensive tertiary hospitals in Changsha, Hunan Province between June 14, 2022 and June 13, 2023, by our trained research assistants who also serve as the leaders of the corresponding wards’ nursing responsibility teams, specifically during the assistants’ duty hours. The following patients were included: (a) patients with pathologically confirmed primary solid carcinoma, staged as II - IV according to the Tumor-Node-Metastasis staging system, (b) patients aware of their cancer diagnosis, (c) patients aged 18 years or older, (d) patients capable of verbal and written communication, and (e) patients who provided consent to participate in the study. These participants were excluded if they had cancer recurrence, a history of psychological disorders or intellectual disabilities, had received psychological treatment within 3 months prior to enrollment, recently taken antidepressant and anti-anxiety drugs, or were critically ill and unable to self-report. Written informed consent was obtained from each participant before formal investigation. In this study, a total of 1050 questionnaires were distributed, and 979 questionnaires were collected, resulting in a response rate of 92.3%. After excluding 8 questionnaires with missing items, 971 valid questionnaires were ultimately determined.

This study was approved by the Institutional Review Board of the Second Xiangya Hospital, Central South University (Approval no. LYG20XX1X0). Eligible participants were given information verbally and a written explanation of the study details to fully understand the aims and procedure of the study and their rights before signing the written informed consent.

### Instrument with validity and reliability

#### General information questionnaire

Demographic data, including age, gender, occupational status, marital status, education level, and information on the primary caregiver, were collected through inquiries or self-reported fill-in methods. Patient information regarding tumor type, Tumor-Node-Metastasis (TNM) staging, and history of radiotherapy was retrieved from the hospital’s electronic medical record system.

#### Mandarin version demoralization scale

The DS-MV comprises five dimensions: loss of meaning (5 items), dysphoria (5 items), disheartenment (5 items), helplessness (4 items), and sense of failure (5 items), amounting to a total of 24 items, utilizing a Likert 5-point rating scale ranging from 0 to 4, subjects are instructed to self-report their state based on the preceding two weeks. The total score of the DS-MV ranges from 0 to 96, with higher scores indicating a higher degree of demoralization. the DS-MV was introduced into China by Hung in 2008. Previous studies have demonstrated satisfactory internal consistency of the DS-MV among Chinese cancer patients. The internal consistency reliability for the overall scale and its subscales (loss of meaning, dysphoria, disheartenment, helplessness, and sense of failure) in this study was acceptable. Cronbach’s α was 0.901 for the overall scale, while the subscales yielded α values of 0.810 (loss of meaning), 0.723 (dysphoria), 0.826 (disheartenment), 0.734 (helplessness), and 0.724 (sense of failure).

### Data collection and analysis

Ahead of the official survey, a pre-survey was conducted with four cancer patients to evaluate the questionnaire’s readability. Consequently, a refined questionnaire instruction and series were developed. During the survey, patients received a comprehensive explanation of the study’s objectives, methods, significance, and completion guidelines from trained assistants. Following informed consent, a paper-based questionnaire was administered, and responses were verified onsite. Uncertain information was clarified with patients to guarantee accuracy. To minimize disruption to their medical routines, data collection occurred 1~2 days after admission, ensuring a smooth and patient-friendly process.

In the study, quantitative data were presented as mean ± SD, while categorical data were described as frequency/percentage. A series of LPA models were iteratively fitted and compared, starting from a single-profile model, to identify the optimal model with the highest fitting index. The model fitting indicators included: (1) Akaike Information Criterion (AIC), Bayesian Information Criterion (BIC), and sample-corrected BIC (aBIC). The smaller the values of AIC, BIC, and aBIC, the better the model fitting. (2) Entropy, ranging from 0 to 1, indicating a more accurate classification as it approaches 1. Generally, Entropy >0.8 indicates that the classification accuracy of the model exceeds 90%. (3) Lo Mendell Rubin Likelihood Ratio Test (LMR) and Bootstrap Based Likelihood Ratio Test (BLRT). When *p* < 0.05, the K-class model outperforms the *K-1* model, determining the optimal fit. The number of latent profiles was decided by combining fit criteria. Cohen’s *d* estimated effect size (0.2=small, 0.5=medium, 0.8=large). A three-step approach considering classification errors was recommended to analyze sample characteristics’ impact on profile memberships (16).

Additionally, we used the robust three-step (R3STEP) method to analyze sociodemographic and disease factors’ predictive effects on profiles. The receiver operating characteristic (ROC) analysis determined the optimal cutoff for the 24-item DS-MV. Classifier performance was assessed using the area under the ROC curve (AUC), sensitivity, specificity, accuracy, and Youden’s index. An AUC value close to 1 indicates high classification accuracy. Optimal cutoff was based on maximum Youden’s index.

To justify the high AUC value, 5-fold cross-validation with a fixed random seed (123) and validation sets generated by 10 different random seeds were performed. Overfitting risk was quantified by comparing the absolute differences of sensitivity and specificity between training and validation sets, with a 5% critical threshold. The Hosmer-Lemeshow goodness-of-fit test was conducted with the sample re-stratified into 5 groups (expected count ≥5 per group) to verify model-data consistency. Sample homogeneity analysis was implemented via stratified ROC analysis by cancer type, stage and age group. Analyses were done in SPSS 26.0, Mplus 8.0, and R 4.0.3. A two-sided *p* <.05 was considered statistically significant.

## Results

Before formal analysis, a Harman’s single-factor test was conducted to assess the common method bias in the scale responses. Five factors with eigenvalues exceeding 1 were extracted, with the primary factor explaining 32.5% of the variance, which is below the conventional critical value of 40.0%. This indicates that there is no significant common method effect in the research data.

### Characteristics of the sample

This study involved 971 participants, aged 18~78 (mean: 53.97 ± 9.83). Mainly women (85.0%) with 825, and men (15.0%) with 146. Of these, 313 (32.2%) were employed, while 658 (67.8%) were not. Majority were married (90.5%), 19 unmarried (2.0%), and 73 divorced/widowed (7.5%). Educationally, 262 (27.0%) completed primary or below, 388 (40.0%) junior high, 187 (19.3%) high school/vocational, and 134 (13.8%) college or above. Regarding informal caregivers (who provided daily care and emotional support for participants), 588 (60.6%) were spouses, 289 (29.8%) children, 32 (3.3%) parents, and 62 (6.4%) other relatives or non-relatives. Cancer types included: 85 (8.8%) digestive, 61 (6.3%) thoracic, 761 (78.4%) reproductive, 44 (4.5%) head/neck, 17 (1.8%) urinary, and 3 (0.3%) bone/soft tissue. TNM staging showed 424 in II (43.7%), 391 in III (40.3%), and 156 in IV (16.1%). 606 participants (62.4%) had received radiotherapy synchronously.

### Statistical overview of the key results

**Table 1** shows the DS-MV scores and dimensions for this group. Due to the helplessness subscale comprising 4 items (while all other subscales of the DS-MV contain 5 items), mean scores (instead of averages) are reported to ensure comparability across dimensions. The group exhibited higher distress in disheartenment and failure, with meaninglessness showing the lowest distress level.

**Table 1:** Assessment scores of DS-MV and each dimension.

Variable	Mean ± SD	Range	Average score of items
DS-MV	31.66 ± 12.19	0~75	1.32
Loss of Meaning	4.63 ± 2.90	0~18	.93
Dysphoria	7.05 ± 3.32	0~18	1.41
Disheartenment	7.66 ± 3.92	0~20	1.53
Helplessness	4.92 ± 2.68	0~16	1.23
Sense of Failure	7.40 ± 3.19	0~20	1.48

DS-MV, Mandarin Version of Demoralization Scale; SD, standard deviation.

### Latent profile analysis results

**Figure 1:**
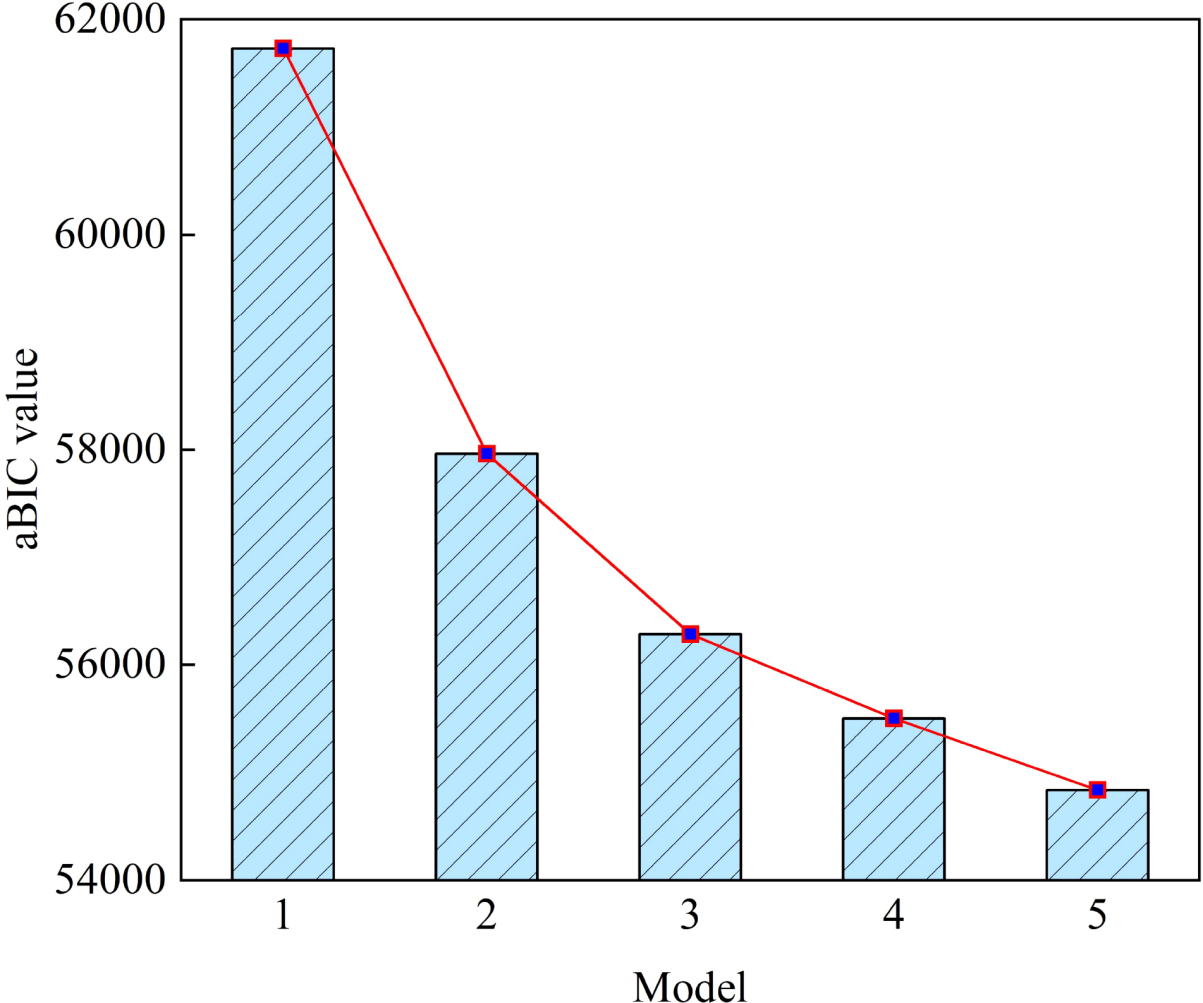
Scree plot of aBIC from LPA. aBIC, adjusted Bayesian Information Criterion; LPA, Latent Profile Analysis.

Starting with a single-profile baseline, we fitted 1~6 potential profile models based on DS-MV scores. **Table 2** shows their fitting indicators. All models had Entropy>0.90, indicating good overall classification reliability, with the three-profile model standing out for its higher entropy and stronger clinical applicability. AIC, BIC, and aBIC decreased with more profiles, while LMR and BLRT of models 1~5 were significant (*p* < 0.05), suggesting that each additional profile statistically improved model fit compared with the previous one. Notably, the 4-profile model showed superior AIC (55290.23) and BIC (55890.27) compared with the 3-profile model (AIC = 56118.24, BIC = 56596.32), with significant LMR (*p* = 0.014) and BLRT (*p* < 0.001). However, AIC and BIC only reflect statistical fit to the dataset and fail to account for classification reliability or clinical practicality—core priorities of our study, which aims to develop a clinically actionable tool. The aBIC scree plot (**Figure 1**) revealed a slowdown from 3 to 4 profiles, marking a clear turning point beyond which adding profiles yielded only marginal fit gains.

**Table 2:** Model fit indices for one- to six-latent profile solutions and corresponding profile prevalence.

Model	AIC	BIC	aBIC	Entropy	LMR	BLRT	Profile prevalence
1	61649.01	61883.17	61730.72	−	−	−	−
2	57837.90	58194.02	57962.17	.915	.000	.000	.660/.340
3	56118.24	56596.32	56285.07	.934	.000	.000	.223/.599/.179
4	55290.23	55890.27	55499.62	.919	.014	.000	.162/.258/.532/.048
5	54587.80	55309.791	54839.74	.920	.000	.000	.149/.434/.120/.250/.047
6	54156.00	54999.95	54450.51	.921	.505	.000	.166/.375/.241/.064/.107/.047

AIC, Akaike Information Criterion; BIC, Bayesian Information Criterion; aBIC, sample-corrected BIC; LMR, Lo Mendell Rubin Likelihood Ratio Test; BLRT, Bootstrap Based Likelihood Ratio Test.

Guided by the fundamental principle of LPA model selection (balancing statistical fit, classification reliability, and clinical interpretability), we selected the three-profile model (Entropy_Model3_ = 0.934) as optimal. Critically, this model classifies subjects into three distinct groups: low-symptom group, moderate-symptom group, and high-symptom group, which perfectly aligns with clinical intervention logic. Specifically, the low-symptom group requires no additional intervention (routine care only), the moderate-symptom group needs targeted psychological support, and the high-symptom group demands psychiatric consultation—fully conforming to the hierarchical intervention needs in clinical practice. The model’s prediction accuracy for C1 (low-symptom), C2 (moderate-symptom), and C3 (high-symptom) reached 97.2%, 97.3%, and 97.4% respectively, with an overall correct prediction rate of 97.3% (945/971 subjects), indicating satisfactory classification performance. In contrast, the 4-profile model included a fourth profile with a prevalence of only 4.8%, which had ambiguous clinical characteristics and would unnecessarily complicate clinical intervention decisions, reducing the scale’s practical utility in routine care.

**Figure 2:**
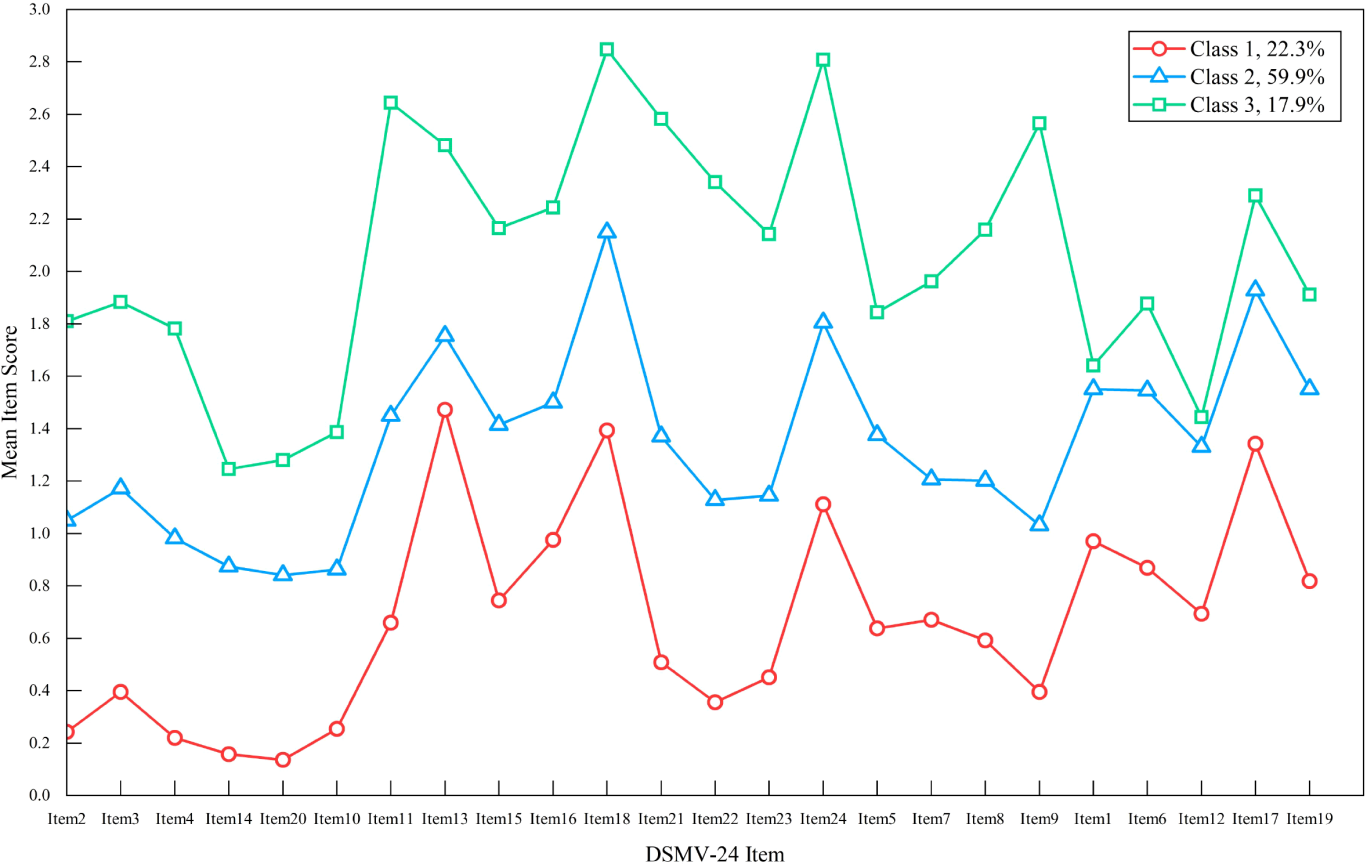
Best-fitting three-class patterns from DS-MV items. DS-MV, Mandarin Version of Demoralization Scale.

We conducted feature analysis and naming for each potential profile by carefully examining the trend chart displaying the average score of each item within each profile of the three-profile model (see **Figure 2**): ① Profile 1, named “minimal demoralization-meaningful” had the lowest scores, especially in meaning loss (avg.<0.4), accounting for 22.3% (n=216). ② Profile 2, “moderate demoralization” scored between Profiles 1 and 3, comprising 59.9% (n=581). ③ Profile 3, “severe demoralization-hopelessness” had the highest scores, especially in helplessness (“I feel hopeless”), accounting for 17.9% (n=174).

Utilizing the three-profile latent class model, **Table 3** displays means, SD, and Cohen’s d for the three profiles, revealing significant differences in their overall mean scores (*p* < 0.05). This validates the chosen model. Consequently, the “moderate demoralization” and “severe demoralization-hopelessness” groups are classified as “cases”, while the “minimal demoralization-meaningful” group is classified as the “non-cases” group. The risk of clinical demoralization among Chinese cancer patients stands at 78.3% (95% CI: 75.7%~80.9%).

**Table 3:** Means, standard deviations, and Cohen’s *d* for the three profiles of DS-MV.

Mean			SD			Cohen’s *d*		
C1	C2	C3	C1&C2	C1&C3	C2&C3	C2: C1	C3: C1	C3: C2
15.64	32.23	49.26	9.32	18.21	9.25	1.78	1.85	1.84

C1 = “minimal demoralization-meaningful” group; C2 = “moderate demoralization” group; C3 = “severe demoralization-hopelessness” group; SD = standard deviation.

### Univariate analysis results

To gain deeper insights into the factors predicting demoralization, this study benchmarked the “minimal demoralization-meaningful” group. Leveraging the R3STEP method nested in LPA, we formulated a regression mixed model encompassing predictive variables. Each variable’s predictive strength was assessed through partial regression coefficients. Our analysis showed that, compared to the benchmark group, work status and synchronous radiation therapy were common positive predictors for both the “moderate demoralization” and “severe demoralization-hopelessness” groups. Conversely, education level negatively predicted for both groups. Notably, TNM staging significantly positively predicted only for the “severe demoralization-hopelessness” group, with statistically significant differences (*p* < 0.001, *p<*0.05), as shown in **Table 4**. In essence, cancer patients in the moderate and severe demoralization groups were more likely to be unemployed or take work breaks, have lower education levels, and undergo synchronous radiotherapy. Furthermore, those in the severe demoralization group were more likely to have a higher TNM staging.

**Table 4:** The correlation between sociodemographic and disease-related variables and each potential category.

Variables	Moderate demoralization			Severe demoralization-hopelessness		
	*B*	*p*	Exp(*B*) (95% CI)	*B*	*p*	Exp(*B*) (95% CI)
Age(continuous)	-.001	.887	1.00(.98~1.02)	.002	.853	1.00(.98~1.03)
Gender(women = ref)	.035	.888	1.04(.64~1.67)	-.465	.226	.63(.30~1.33)
Occupational status(at work = ref)	.781	<.001	2.18(1.54~3.10)	.991	<.001	2.69(1.62~4.47)
Marital status (married = ref)	.001	.997	1.00(.70~1.43)	.265	.206	1.30(.87~1.96)
Education (primary school or below = ref)	-.362	<.001	0.70(.59~0.83)	-.927	<.001	.40(.31~0.51)
primary caregiver(spouse = ref)	.055	.616	1.06(.85~1.31)	.130	.353	1.14(.87~1.50)
Cancer type(gastrointestinal neoplasms = ref)	-.035	.757	.97(.78~1.20)	.096	.565	1.10(.79~1.53)
TNM stage(II = ref)	-.136	.283	0.87(.68~1.12)	.428	.008	1.54(1.12~2.11)
Simultaneous radiation therapy(no = ref)	.396	.028	1.49(1.04~2.12)	.858	.001	2.36(1.43~3.90)

### Receiver operating characteristic analysis results

**Figure 3:**
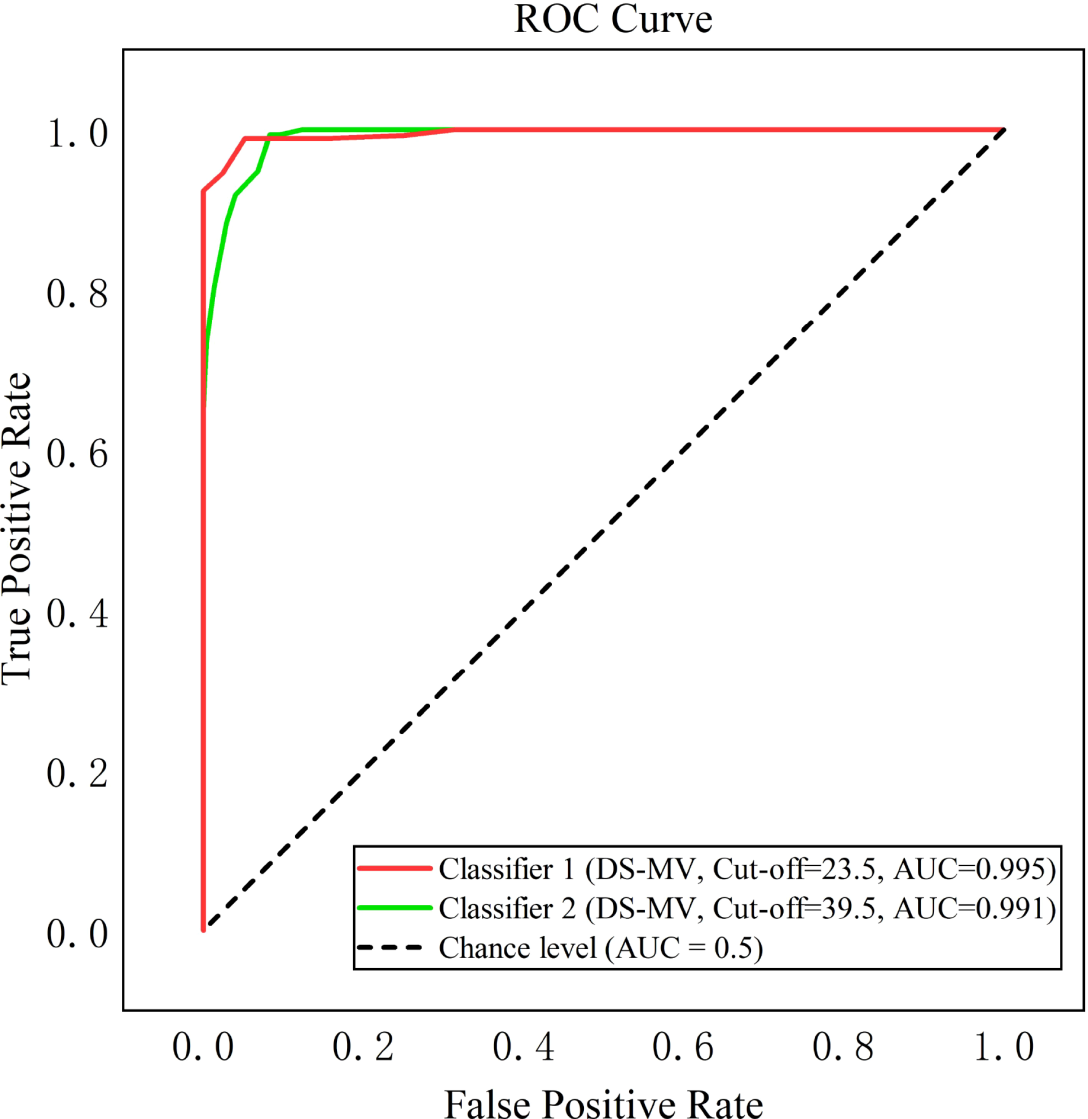
ROC curves for DS-MV demoralization screening. ROC, Receiver Operating Characteristic; DS-MV, Mandarin Version of Demoralization Scale.

To eliminate the potential circularity between LPA-derived groups and ROC validation, the definition of “cases/non-cases” in ROC analysis was determined based on clinical intervention indications and confirmed by expert demonstration, which was completely independent of LPA results. Initially, individuals assigned to the “minimal demoralization” group were defined as “non-cases”, and the remaining were viewed as “cases”. Based on the binary outcome, the ROC curve (**Figure 3**, Classifier 1) was plotted for the DS-MV, with an AUC value of 0.995 (95% CI: 0.993~0.998) indicating strong classification accuracy for demoralization syndrome. The optimal cut-off threshold of 23.5, determined by the Youden Index (0.937), yielded sensitivity, specificity, and accuracy of 98.9%, 94.8%, and 93.7%, respectively.

Correspondingly, the optimal predicted probability cut-off (P_cut) was 0.54 (rounded from the original 0.5395614) based on the maximum Youden Index (0.937), used to classify training/validation sets (≤0.54→0 [“non-cases”], >0.54→1 [“cases”]). Sensitivity analyses confirmed the high AUC stability: 5-fold cross-validation (random seed=123) yielded a training set AUC of 0.995 ± 0.001, while validation sets (10 random seeds: 159, 361, 456, 507, 671, 719, 789, 842, 934, 1011) had an AUC of 0.996 ± 0.001, indicating high consistency between sets. The training set had mean sensitivity/specificity of 98.9%/94.8%, and the validation set 98.9%/94.9%; the absolute differences (0.0% for sensitivity, 0.1% for specificity) were far below the 5% overfitting threshold, confirming minimal overfitting and strong generalizability to new data. The Hosmer-Lemeshow goodness-of-fit test (5-group re-stratification) further validated model adaptability (*χ²* = 4.533, *df* = 3, *p* = 0.606>0.05), demonstrating consistency between predicted probabilities and actual data distribution without model specification-related systematic bias.

Sample homogeneity analysis ruled out single-subgroup-driven high AUC: stratified ROC analyses showed all subgroups had AUCs ranging.991–.999. For cancer type: reproductive system cancer (n=761, AUC = 0.996, 95% CI: 0.994~0.999) and other cancers (n=210, AUC = 0.992, 95% CI: 0.985~1.000); for stage: II (n=424, AUC = 0.994, 95% CI: 0.990~0.999), III (n=391, AUC = 0.999, 95% CI: 0.997~1.000), IV (n=156, AUC = 0.991, 95% CI: 0.980~1.000); for age: <50 years (n=279, AUC = 0.998, 95% CI: 0.996~1.000) and ≥50 years (n=692, AUC = 0.994, 95% CI: 0.991~0.998); for gender: Men (n=146, AUC = 0.992, 95% CI: 0.982~1.000) and Women (n=825, AUC = 0.996, 95% CI: 0.994~0.999). Small AUC fluctuations across subgroups confirmed the DS-MV’s stable diagnostic performance.

To enhance early warning for severe demoralization among cancer patients, we redefined the “severe demoralization-hopelessness” group as “cases” and labeled all other patients as “non-cases.” The DS-MV’s ROC curve demonstrated a robust AUC of 0.991 (95% CI: 0.987~0.995), indicating significant predictive capability (**Figure 3**, Classifier 2). The optimal cut-off threshold was determined to be 39.5, aligning with a Youden index of 0.911. At this threshold, the sensitivity and specificity achieved were 99.4% and 91.7% respectively. the ROC curve.

## Discussion

As far as we know, especially among Chinese cancer patients, this study stands out as one of the few that employs LPA to classify demoralization syndromes. Six LPA models underwent testing, and a three-profile model proved optimal, as determined by fit indices and scree plot analysis. This model demonstrated significant discrimination in latent profile probabilities, highlighting the heterogeneity within the sample regarding demoralization syndrome. Cohen’s d values exceeding 0.8 further confirm the accuracy of the classification.

Based on the responses to DS-MV items, the three profiles were categorized as: “minimal demoralization-meaningful” (22.3%), “moderate demoralization” (59.9%), and “severe demoralization-hopelessness” (17.9%). The latter group was identified as “cases”, while the others were “non-cases”. A risk assessment indicated that 78.3% (95% CI: 75.7%~80.9%) of Chinese cancer patients were prone to symptoms of demoralization syndrome, slightly exceeding previous findings by Wu et al. (7) which reported a 75.7% rate using the DS-II simplified version. A possible explanation for our survey results is that cancer imposes a prolonged, multi-faceted burden on patients. They are prone to a perceived inability to cope due to difficulties and delays in adapting to the disease, manifesting in symptoms like poor sleep, appetite loss, fatigue, and suicidal thoughts/behaviors. The variance in findings could stem from different scales, geographical/temporal variations, and the advanced cancer stages in our study (excluding stage I).

Observations showed consistent scoring patterns across the DS-MV items, with prominent peaks in average scores for items 1, 13, 17, 18, and 24. These peaks reflect a sense of failure, dysphoria, and disheartenment. This indicates that initial demoralization symptoms in Chinese cancer patients include dysphoria, disheartenment, and a sense of failure. These insights can aid clinical recognition and targeted interventions.

The “minimal demoralization-meaningful” group exhibited the lowest mean scores in all DS-MV items, averaging at 15.64 ± 6.88. Notably, the five items representing loss of meaning had the lowest scores (see **Figure 2**), indicating minimal risk. Conversely, the “moderate demoralization” group had higher mean scores in all indicators, with an overall average of 32.23 ± 5.31, which was significantly higher than the minimal group, highlighting profile differences. A key finding was the peak score in item 18: “I feel distressed about what is happening to me”. Psychological distress deemed as the sixth vital sign in cancer patients (17), ranges from vulnerabilities, sadness, and fear to anxiety, depression, and psychological crises. It is considered a key factor affecting cancer progression and patients’ quality of life (18). Drawing from the stress and coping paradigm (19), it urges patients and caregivers to promptly recognize moderate demoralization. Tailored interventions like cognitive evaluation, social support, and coping methods, customized to individual personalities and experiences, are recommended to alleviate negative emotions and boost self-efficacy (20), thus mitigating this specific psychological distress. The “severe demoralization-hopelessness” group had a significantly higher overall DS-MV mean score of 49.26 ± 7.49. Notably, item 9 (“I feel hopeless”), which assesses hopelessness, reached a peak. Although hopelessness is conceptually distinct from helplessness (the latter reflecting inability to cope), disheartenment, and dysphoria, this item is categorized under the helplessness dimension in DS-MV. In Chinese culture, such an expression of hopelessness may be interpreted as suicidal ideation, reflecting a severe negative psychological state. It underscores the importance of clinical staff promptly identifying this subgroup and offering timely guidance on coping strategies. If necessary, referral to a psychological expert is recommended. The smallest differences between the “moderate” and “severe” groups were observed in items related to the sense of failure dimension, specifically items 1 and 12 (item 1: “There is a lot of value in what I can offer others”; item 12: “I cope fairly well with life”). Both items are reverse-scored in the DS-MV, which explicitly requires participants to reverse their response logic compared to forward-scored items within the same dimension. This performance was viewed as potential response errors in Likert reverse-scored items (21), indicating a “patterned responses” effect (16) rather than a pure coincidence or a definitive evaluation. Patients in the “severe demoralization-hopelessness” group may have exhibited patterned responding. They intended negative responses but answered incorrectly on two reverse-scored items.

Despite the challenge of intervening in demographic and disease factors, exploring them can aid in early identification of high-risk demoralized patients, guiding clinical interventions. The R3STEP method revealed work status, education, tumor stage, and radiotherapy status as predictors of cancer patient profiles. This aligns with Lee et al.’s findings on the predictive role of work status (4). Comprehensive cancer treatments, including surgery, chemotherapy, and radiotherapy, often prevent patients from working. Unlike freelancers or employed individuals, patients on leave often face economic hardships and limited social support. Some patients rely on relatives or debt, heightening hopelessness and low self-esteem, leading to demoralization. Previous studies show that cancer patients’ social, occupational, and economic roles contribute to a sense of health, value, and social connectedness (22). This study also found that low education increases the risk of demoralization, aligning with previous research (5, 23). Educational level is frequently intimately linked to individual psychological adjustment, the quality of life, etc. A higher educational level endows individuals with a greater array of effective resources, such as economic support, health literacy support, etc. to cope with diseases, particularly life-threatening diseases. Our study concurs with Vehling et al. (24) that tumor staging is a predictor of demoralization, with later stages linked to higher demoralization scores. Tumor staging correlates with treatment, symptoms, prognosis, and recurrence, and as it advances, so do care needs, physical and mental burdens, and economic strain, often leading to feelings of hopelessness and despair. However, some studies have not found a significant link between demoralization and tumor staging (25). Instead, these studies suggest that cancer patients face significant physical and psychological challenges from the time of diagnosis. The fact of having cancer itself brings the realization of a life countdown, limited life span, which is accompanied by anxiety, fear of recurrence, and other mental health challenges.

These challenges persist regardless of the tumor’s stage, highlighting the importance of providing comprehensive support to cancer patients throughout their illness journey. Finally, this study has also revealed that receiving synchronous radiotherapy increases the level of demoralization in cancer patients, aligning with the findings of Robinson et al. (26) (the level of demoralization was higher in radiotherapy patients compared to non-radiotherapy patients, with a *Z* score of -1.99 and a *p* value of 0.047). Analyzing the reasons, it is observed that, on one hand, patients receiving synchronous radiotherapy may have more severe conditions, and on the other hand, the side effects of radiotherapy may further exacerbate the cancer-related symptom burden (27), ultimately leading to an increase risk of demoralization.

The Demoralization Scale (DS) is a widely used tool for screening demoralization in clinical settings, with translations into multiple languages and broad cross-cultural applicability. This study adopted LPA and ROC curve analysis to establish the optimal cut-off threshold for the 24-item DS-MV, aiming to enhance its localization, application, and guidance for research and clinical interventions in Chinese cancer populations. Our findings recommend a threshold of 23.5, differing from previous domestic and international studies (6, 7), and with superior performance metrics, including AUC of 0.995, Youden Index of 0.937, sensitivity of 0.989, specificity of 0.948, and accuracy of 0.937. We attributed these discrepancies in diagnostic thresholds to variations in reference standards, clinical environments, comorbidities, and cultural backgrounds. Unlike prior studies using structured interviews, our approach efficiently classifies patients into “cases” and “non-cases” based on LPA results. This objective classification facilitates the identification of patients with moderate or severe demoralization, enabling timely interventions.

Additionally, using the same methodology, we identified a critical score of 39.5 for severe demoralization. This score not only further underscores the diagnostic precision of the DS-MV but also facilitates the urgent referral of patients to psychological experts for intervention. Supported by robust diagnostic metrics, including an AUC of 0.991, a Youden Index of 0.911, a sensitivity of 0.994, and a specificity of 0.917, this threshold effectively identifies the high-risk “severe demoralization-hopelessness” group (e.g., those with treatment non-adherence or suicidal ideation).

Notably, a review of prior demoralization scale validation studies reveals a critical research gap: most only reported cut-off thresholds and basic diagnostic indicators (e.g., sensitivity, specificity) but omitted AUC values, which are indispensable for assessing overall discriminative ability. According to our retrieved literature data, only one study on the Italian Demoralization Scale (DS-IT) in kidney transplant recipients reported an AUC of 0.92 (28). This result is recognized as “satisfactory” for reliably differentiating demoralized individuals. Even against this benchmark, the DS-MV still achieved a substantially higher AUC of 0.995. The main potential reasons for the high AUC value observed in this study can be summarized as follows: First, the study included a robust sample of 971 patients. This sample size far exceeds the minimum requirement for validating clinical screening tools and approaches the large-sample threshold needed to optimize the reliability of prediction models. It effectively mitigates random measurement errors and overfitting risks, thereby ensuring the accuracy of AUC estimates. Second, the DS-MV underwent rigorous Mandarin localization. This revision reduces response bias among participants and enhances the scale’s measurement precision. Third, the study sample primarily consists of hospitalized cancer patients. These patients exhibit more prominent and persistent demoralization symptoms due to acute stressors such as disease progression and invasive treatments, which results in a high signal-to-noise ratio in the research data. Collectively, these findings confirm the DS-MV as a reliable, culturally adapted tool for demoralization screening among Chinese hospitalized cancer patients.

The current study has some limitations. Firstly, despite using multiple objective indices, the best-fitting model couldn’t be conclusively determined, relying instead on a semi-subjective assessment of the aBIC scree plot, potentially affecting the study’s credibility. Hence, future replication is needed. Secondly, LPA, an exploratory technique based on posterior probabilities, risks misclassification if group distributions are even. However, our profiles showed strong discrimination. Thirdly, while the DS-MV’s optimal cut-off was analytically determined, it’s not a substitute for a clinical psychiatric interview. Thus, these findings should be interpreted with caution pending further research. Fourthly, as a cross-sectional study, it cannot infer causal relationships or track temporal changes. Future research may adopt longitudinal designs to establish temporal associations. Fifthly, generalizability is limited by single geographic sampling (two tertiary hospitals) and department-specific recruitment, leading to overrepresentation of females and reproductive system cancer patients. Subsequent studies should adopt multi-center designs, expand to diverse ethnicities and socioeconomic backgrounds, and use stratified sampling to align subgroup proportions with clinical practice. Sixthly, self-reported measures carry response bias risks (e.g., acquiescence, patterned responding). Future research could supplement with clinician-rated scales or validity checks to enhance reliability. Seventhly, LPA-derived “cases” lack clinical validation, limiting interpretability. Future studies should validate profiles against established criteria to strengthen clinical relevance. Eighthly, Although the exclusion of Stage I patients in this study is based on the core application scenario of the original scale, the research need to avoid the dilution of demoralization symptom signals, and the ethical considerations of reducing patients’ psychological burden, there remains the possibility that it may inflate the prevalence of demoralization and alter its class structure. Future studies may consider establishing corresponding clinically applicable cutoff values for patients at different cancer stages.

## Conclusions

The study delved into the demoralization experiences of Chinese cancer patients, identifying three profiles: minimal yet meaningful, moderate, and severe due to hopelessness. Investigating the associated factors helps identify high-risk groups earlier and tailor precision interventions. Our R3STEP method revealed work/education status, tumor stage, and radiotherapy’s impact on demoralization. Moreover, by combining LPA and ROC curve analysis, we identified an optimal 23.5 cut-off for the 24-item DS-MV, exceeding previous standards in accuracy, sensitivity, and specificity. This LPA-based categorization is efficient and cost-effective. For clinical practice, we recommend the following operational rule: a total DS-MV score≥24 points is defined as clinically significant demoralization, while a score ≤ 23 points is considered non-significant. We also found a threshold of 39.5 for severe demoralization, aiding clinicians in referring patients to psychologists. This underscores the importance of integrating psychological care into cancer treatment, especially in cross-cultural contexts.

## Data Availability

The raw data supporting the conclusions of this article will be made available by the authors, without undue reservation.
